# Using solid-phase microextraction during ultrasound reveals higher aqueous PAHs release from contaminated sediment

**DOI:** 10.1016/j.ultsonch.2022.105981

**Published:** 2022-03-12

**Authors:** Danielle S. Kohan, Roman P. Lanno, Linda K. Weavers

**Affiliations:** a Environmental Science Graduate Program, The Ohio State University, Columbus, OH 43210, United States; b Department of Evolutionary Ecology and Organismal Biology, The Ohio State University, Columbus, OH 43210, United States; c Department of Civil, Environmental, and Geodetic Engineering, The Ohio State University, Columbus, OH 43210, United States

**Keywords:** Sediment remediation, Contaminant desorption, SPME passive sampling, In-situ ultrasonic remediation, In-situ sampling

## Abstract

Ultrasound (US) releases polycyclic aromatic hydrocarbons (PAHs) from contaminated creosote sediments and degrades PAHs in aqueous solution. However, it is unclear how much PAHs release occurs during active US compared to after US is stopped. In this study, solid-phase microextraction (SPME) was used to determine aqueous PAH concentrations desorbed from Little Scioto River creosote contaminated sediment during exposure to 20 kHz ultrasound (US) at a power of 430 W L^−1^. First, SPME fiber-water partition coefficients,$K_{\mathrm{SPME}}$, were experimentally determined and shown to be comparable with previous studies. Next, PAH concentrations released into aqueous solution were determined by sequentially exposing fresh, conditioned SPME fibers in a reactor containing the contaminated sediment and DI for 10 min periods. Three consecutive 10 min periods each were measured during US and after US. Compared to mixing only, PAHs desorbed during ultrasound was significantly higher. In addition, for phenanthrene, anthracene, and fluoranthene, US showed significantly higher aqueous concentrations during US compared with after US. For these less soluble PAHs, desorption into the aqueous phase reaches and surpasses expected equilibrium aqueous concentrations. However, when US is ceased, PAHs appear to resorb onto sediment resulting in the lower concentrations measured in these PAH compounds after US that are similar to expected equilibrium aqueous concentrations. Typical analytical extraction methods for determining the effects of US require stopping treatment and may underestimate the potential US has for release of contaminants from sediment during US. Using SPME during US treatment reveals that the concentration of PAHs during US may surpass the expected equilibrium aqueous concentration of some PAHs congeners. To our knowledge, this higher concentration observed during US compared to after US has not been shown previously.

## Highlights

• [PAHs]_aq_ during US is higher than [PAHs]_aq_ after US is ceased.
• Analysis of [PAHs] after US treatment is ceased underestimates released PAHs.
• [PAHs] resorb onto sediment within 10 min after US is turned off.
• SPME fibers allow for measurement of [PAHs]_aq_ during course of US treatment.

## 1 Introduction

Polycyclic aromatic hydrocarbons (PAHs), a common class of sediment pollutant, persist in many locations around the United States and worldwide. One legacy source of PAHs contamination of sediments results from effluent emanating from wood-treating facilities that produce creosote, a viscous, oily liquid comprised of a mixture of hydrocarbons created from coal tar. The primary strategy for cleaning up PAHs contaminated sediment is an *ex-situ* approach: dredging the sediment followed by disposal or treatment. Developing *in-situ* remediation technologies that do not require dredging may be more cost effective and incur fewer environmental impacts. Currently, these strategies have barriers including technological limitations, unclear oxidant demand, challenges with mass transfer of the contaminants from sediment to the aqueous phase where treatment occurs and cost efficiency. At present, these barriers eliminate in-situ alternatives as realistic options.

Ultrasonic remediation has potential as an *in-situ* approach that does not require any chemical additives [2], [15]. Ultrasound works via acoustic cavitation. Microbubbles, influenced by the sound wave, grow by rectified diffusion and eventually become unstable and implode. At collapse, the cavitation bubbles produce tremendous pressure and heat in the bubble while shock waves emanate from the bubble wall. This violent collapse erodes and fractures sediment grains present near the bubbles [32], [43], releasing bound contaminants and accelerating mass transfer of contaminants from the sediment particles into the aqueous phase [15], [29], [30], [52]. The high temperatures also result in bond dissociation of water, forming •OH radicals which are capable of degrading contaminants through oxidation pathways [17].

Solid phase micro extraction (SPME) is a passive sampling technique to measure aqueous phase organic contaminants. This passive sampling technique is considered non-depletive; it does not significantly change the concentration of solute in a solution. There are different devices that can be used to accomplish this measurement, but the general method is the same. A small portion of the aqueous phase contaminant sorbs to the extraction material. The mass sorbed is related to the aqueous concentration by a partition coefficient, K_SPME_, an empirically-determined, compound-specific parameter used to quantify aqueous concentrations. SPME offers the advantage of only quantifying dissolved PAHs, not PAHs bound to sediment or to colloidal material, a challenging objective when using total exhaustive extraction methods [27], [59].

The objective of this work was to measure the aqueous phase PAH concentrations during ultrasonic (US) exposure to explore the mass transfer of contaminants between the sediment and the aqueous phase *during* and *after* ultrasound. Past work has shown that ultrasonic treatment reduces sediment particle size [37], [43] and accelerates the rate of desorption of contaminants from solids [5], [8], [20], [31], [52]. Also, it has been shown that ultrasound alters adsorption isotherms [30], [31]. Bulk mineral phases are unaltered by ultrasound [22], [52] yet releases of high levels of organic matter that decrease with increasing sonication time [43] and generation supersaturated concentrations of metal ions from mineral oxide [22] are observed. Typically, measurement occurs after US has concluded, and techniques require separation of solids from the aqueous phase through techniques such as filtration and centrifugation prior to aqueous concentration quantification. Aqueous PAH concentrations *during* sonication may not be at equilibrium, an effect that could only be measured if a quantitative tool is used during ultrasonic treatment.

This study uses SPME fibers during US treatment to determine PAH concentrations in solution to measure how the aqueous phase PAH concentrations respond to the presence of ultrasound. Fibers were deployed sequentially to create a timeline of concentration response behavior at various points within a continuous treatment time. Measuring the effect US has on the relationship between PAHs mass bound to the sediment and aqueous PAH concentrations *during* treatment provides key information about the change in concentrations while US is occurring compared to after ultrasound is ceased. This information is necessary to design a practical ultrasonic *in-situ* environmental remediation strategy.

## 2 Materials and methods

### 2.1 Reagents

A mixture of the 16 EPA priority PAHs dissolved in acetonitrile was obtained from Sigma-Aldrich. Methanol (Fisher-Scientific, HPLC grade), acetone (Fisher-Scientific, HPLC grade), hexane (Fisher-Scientific, HPLC grade), acetonitrile (Fisher-Scientific, HPLC grade), and toluene (Fisher-Scientific, HPLC grade) were used as received. Phenanthrene-d10, neat, (Supelco, HPLC grade) was dissolved in a 1:1 mixture of hexane and acetone for use as an instrument internal standard. Pyrene-d10, 500 µg/mL in acetone (Ultra Scientific) was used as a surrogate standard in total sediment extractions. DI water from a MilliQ water purification system was used in all experiments.

### 2.2 Sediment collection and characterization

Freshwater sediment was collected on October 19, 2015 from a contaminated stretch of the Little Scioto river (40.573160, −83.203324) in Marion, OH. Sediment was collected using a push-tube core sampler reaching approximately 60 cm from the sediment surface. After removal, deeper layers of the core were placed into a clean aluminum pan, prior to sub-sampling into 950 mL amber glass jars. Samples were sieved with a 0.312 cm sieve to remove large debris. Sediment was stored wet in the original collection jars at 4 °C until use. Prior to experimentation, samples were homogenized using a rotating tumbler for at least 30 min. The organic carbon content of the collected sediment was analyzed using subtraction from the total carbon (TC) content with a VARIO Max Cube Carbon – Nitrogen Analyzer (STAR Labs, OSU Wooster, OH). The dry weight of the sediments was determined using EPA method 3550C [36].

### 2.3 SPME fiber construction and preparation

Blunt end stainless steel needles were obtained from Hamilton. SPME fibers with a glass core and a 30 µm thick polydimethylsiloxane coating (Polymicro Technologies, Inc.) were cut into pieces 1.91 cm long and conditioned in methanol for 10 min [33], [34]. Double Bubble epoxy (Hardman) was applied to 0.64 cm of the fiber tip, prior to insertion into the needle, leaving 1.27 cm of fiber exposed. These fibers were allowed to set overnight before use. Newly constructed fibers were prepared for experiments through one cycle of exposure to aqueous PAH solution and were additionally conditioned before every experiment. Reproducibility and durability controls are detailed in SI.

### 2.4 Experimental setup

**Fig. 1:**
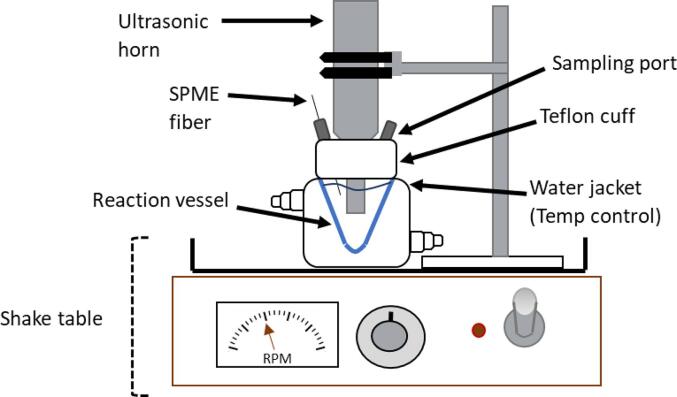
Experimental set up.

A 20-kHz direct immersion ultrasonic probe (model 550, Fisher Scientific) with a 1.2 cm^2^ tip was used in all ultrasound experiments. At an amplitude setting 5.5, the power intensity measured through calorimetry was determined to be 431 WL^−1^. A 60 mL glass conical reactor with a water jacket was used to maintain a constant temperature of 32 ± 3 °C in the reactor. See S.I. for temperature control details. A Teflon collar with 4 sampling ports was fitted to tightly seal the glass reactor to the ultrasonic probe. Two of the four sampling ports in the Teflon collar were fitted with stainless steel fittings to hold the SPME fibers in place during experiments. The entire assembly (ultrasonic probe and reactor) was placed on a shake table (New Brunswick Scientific) set to 125 RPM to ensure mixing throughout both ultrasound and mixing only experiments. Experimental set up is shown in Fig. 1. For all experiments using sediment, 40 mL of DI water was added to 1.2 g sediment. In the absence of sediment, aqueous PAH solutions were created with a PAHs standard in acetonitrile and DI water.

### 2.5 Fiber and total sediment extraction and PAHs analysis

Before each set of experiments involving exposure of a fiber to a sample which contained PAHs, the fiber was prepared by exposure to three serial aliquots of 5 mL of 1:1 hexane/acetone to ensure it was clean before exposure to PAHs. After exposure to PAHs, the fibers were exposed again to three serial aliquots of 5 mL of 1:1 hexane/acetone for analysis. The first two were mixed together for a final sample volume of 10 mL; the final 5 mL aliquot served as a carryover check to confirm the first two desorptions extracted all PAHs from the fiber (see SI for details). Throughout experiments, <10% PAHs were detected in carryover samples.

Extractions to quantify PAH concentrations in the sediment were performed before and after US treatment experiments. EPA method 3550C for med/high predicted contamination range was used [36] with toluene as the extraction solvent [50]. Pyrene-d10 was used as the surrogate standard. A portion (0.3 mL) of the sample extract was recovered and diluted for analysis. A liquid–liquid extraction (3:1 aqueous to toluene) was performed on the supernatant water added to the sediment during the treatment.

Quantitative analysis of PAHs was performed with a gas chromatograph with an ion trap mass spectrometer (ThermoQuest TraceGC, Finnigan Polaris Q) equipped with an autosampler (CTC Analytics Combi Pal) and DB-5MS column (30 m × 0.25 mm × 0.25 µm) operated in splitless mode with transfer line and ion source at 300 °C and 250 °C, respectively. Data was acquired in full scan mode (mass range: 100–350 *m*/*z*). Analytes of interest were identified by searching for the mass of each compound and matching the retention times of each compound with the retention times in the calibration standards. Calibration standards in the expected range of PAHs were created from stock solution and were used for analysis of all quantified PAHs. A five-point calibration curve was created using concentrations of 1, 0.8, 0.6, 0.3, and 0.1 µg/mL standard stock solution. Phenanthrene-d10 was used as an internal standard in all samples and as a calibration standard. Linear regression was used with measured correlation coefficients between 0.98 and 0.99. Limit of detection (LOD) and limit of quantification (LOQ) were estimated as clear peaks with a signal to noise ratio of 3 and 10, respectively. Data was acquired and analyzed using Xcalibur (Thermo Fisher). Example calibration curve and chromatograms are in S.I.

### 2.6 Statistical analysis

Statistical analysis was performed using SPSS Statistics (IBM) and Microsoft Excel. In Table 3, the p values were determined through a multiple comparisons ANOVA (Tukey HSD), a=0.05. Other statistical analysis was performed using the Analysis Toolpak add in for Microsoft Excel (Version 2102).

## 3 Results and discussion

### 3.1 Sediment characterization

First, the sediment was characterized as shown in Table 1. Of the 16 EPA designated PAHs, 9 were quantified. As this legacy contamination has undergone the weathering processes, the lighter molecular weight, more soluble PAHs were detected in a relatively lower concentration compared to the higher molecular weight PAHs. Notably, naphthalene was not quantified in any of the total extractions. Its absence is most likely due to its relatively high volatility, resulting in naphthalene escape over the years during sediment aging in the Little Scioto River. The presence and concentration of PAHs varies among sites contaminated with PAHs from creosote. The total measured sediment PAH concentrations at the Colonial Creosoting Co. Superfund site in Washington Parish, LA, reached 150 mgkg^−1^, whereas higher levels of total PAHs (20000–70000 mgkg^−1^) have been sampled at the Federal Creosote Superfund site in Manville, NJ [53], [54]. A comparison of PAH concentrations in the Little Scioto sediments shows great variability along the contaminated reach of river, indicating that PAHs contamination will vary [35]. Generally, the levels detected are within expected ranges for creosote contaminated sediment sites [35], [38].

**Table 1:** Physical properties and PAH concentrations of sediment collected from the Little Scioto River (Marion, OH). Concentrations represent an average of 3 separate measurements and the RSD.

Sediment Characteristic	Value
% organic carbon	7.4%±0.5%
% dry weight	59.7%±0.3%
Naphthalene, mgkg^−1^	N.D.
Acenaphthylene, mgkg^−1^	55 ± 6
Acenaphthene, mgkg^−1^	70 ± 10
Fluorene, mgkg^−1^	70 ± 20
Phenanthrene, mgkg^−1^	210 ± 30
Anthracene, mgkg^−1^	340 ± 60
Fluoranthene, mgkg^−1^	680 ± 70
Pyrene, mgkg^−1^	760 ± 80
Benzo[a]anthracene, mgkg^−1^	400 ± 50
Chrysene, mgkg^−1^	480 ± 70
N.D. = Not detected	

### 3.2 PAHs equilibrium and partitioning

SPME works through partitioning of analytes between the fiber coating and the aqueous phase of the sample matrix. Fibers with a non-polar, polydimethylsiloxane (PDMS) coating have been widely used for PAHs analysis [9]. At equilibrium, the amount of analyte extracted on the fiber is directly related to the concentration of the sample [60]. Time to equilibrium is affected by several parameters, including fiber type, coating thickness, physical–chemical characteristics of analyte and matrix effects. PAHs reach equilibrium with the SPME fiber more quickly in aqueous solution than in the presence of sediment, an effect which may be attributed to matrix effects in the sediment. For example, organic matter present in sediment, may interfere by competing with the SPME fiber coating for PAHs sorption [7], [19]. Moreover, it has been documented that as PAHs increase in molecular weight, diffusion from the water to the fiber is slower [26]. In other words, lower molecular weight PAHs will reach equilibrium more quickly than higher molecular weight PAHs.

**Fig. 2:**
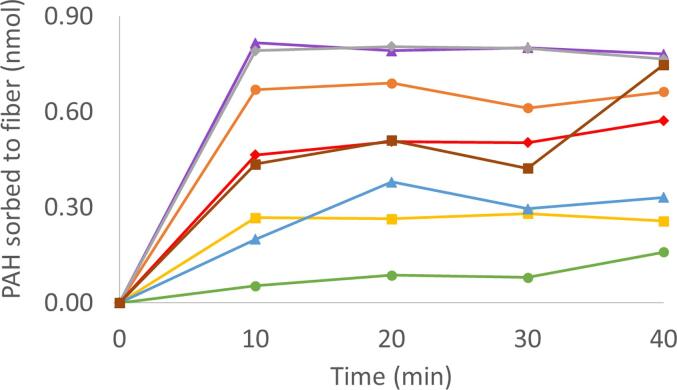
PAH congener sorption to SPME fiber over time. Fresh fiber exposed to a 0.5 µg mL^−1^ aqueous PAH solution. Fiber exposed under mixing conditions only.

Because SPME is an equilibrium method, we determined the time needed to reach equilibrium between various PAHs and the SPME fiber. Fresh, conditioned fibers were exposed to aqueous mixtures with PAHs concentrations of 0.5 µg mL^−1^. The resulting concentrations which sorbed to the fiber are shown in Fig. 2. The low and moderate molecular weight PAHs appeared to sorb onto the fibers rapidly during the first 10 min and appear to remain relatively constant throughout the remainder of the experiment. The plateau observed after 10 min of fiber-solution contact, indicates that the concentration quantified by the fiber is no longer time dependent, indicating equilibrium. Therefore, these PAHs appear to approach and reach equilibrium with the fiber. At equilibrium, the amount on the fiber is directly proportional to the amount in the water [41].

However, for higher molecular weight PAHs, such as anthracene, fluoranthene and pyrene, equilibrium may not have been reached, shown through the apparent increase in quantified concentrations between 30 and 40 min in Fig. 2. This result is consistent with previous SPME fiber equilibrium experiments, which have shown that higher molecular weight PAHs need longer contact times in order to reach equilibrium. Doong et al. [12] observed an equilibration time on the order of several hours, which is an unrealistic equilibration time for a feasible experiment in our study.

Benzo[a]anthracene and chrysene, were not detected, most likely a limitation of the 30 µm PDMS fiber coating. It has been shown that fiber coating thickness and type affect the concentration of PAHs which can sorb to the fiber coating. While the 30 µm PDMS coated fiber is moderately efficient at extraction of the lower to middle range molecular weight PAHs, it is unable to efficiently and reliably extract higher molecular weight PAHs [12], [56]. PAHs of increasing molecular weight up to pyrene were reliably quantified, but the higher molecular weight PAHs larger than pyrene were unable to be quantified. Therefore, we used a contact time of 10 min in subsequent experiments.

Next, we determined equilibrium partition coefficients between the fiber and aqueous solution at 10 min exposure. When using SPME, equilibrium is used to define the partition coefficient between stationary and aqueous phases [10] as calculated using Eq. (1):

(1) $$K_{\mathrm{SPME}}=\frac{n}{C_{e}V_{f}}$$

where n = total moles on the SPME fiber, C_e_ = equilibrium concentration (mol mL^−1^), V_f_ = volume of fiber coating (mL). The concentration that sorbs to a particular SPME fiber varies dependent on matrix effects and type and thickness of fiber coating. Therefore, these partition coefficients are experimentally derived for each specific test system. In our experiments, we determined sorption at 10 min and refer to the partition coefficient as K_SPME10_, recognizing that equilibrium may not be achieved for all PAHs.

**Fig. 3:**
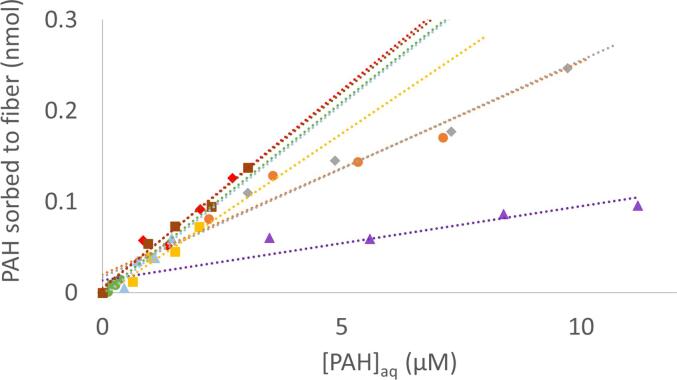
Amount of PAH sorbed to fiber at different concentrations of PAH in aqueous solution. SPME contact with PAH solutions is 10 min (t = 10 min).

In this study, K_SPME10_ was established by exposing fresh fibers to separate aqueous solutions of increasing PAHs concentration. The number of moles, *n,* sorbed onto the SPME fiber after a 10 min contact time, at different aqueous concentrations, was determined and related to C_e_, as shown in Fig. 3. As expected, there was an observed linear increase in sorbed concentration with increasing aqueous concentration for each PAH congener. All PAHs in Fig. 3 were linear, with r^2^ values ranging from 0.88 to 0.98 as shown in Table 2. Thus, K_SPME10_ for each PAH congener was determined from the slopes in Fig. 3 and using Eq. (1). Calculated $K_{SPME10}$ values are reported in Table 2.

**Table 2:** Experimentally determined partition coefficients from Fig. 3 (*m =* slope; r^2^ = coefficient of determination). Reported values with similar SPME fibers of similar composition and thickness.

PAH congener	*m*	r^2^	p-value	logK_SPME_ 30-µm PDMS^a^	logK_SPME_ 100-µm PDMS^b^
Naphthalene	0.08	0.89	0.02	2.68	3.02
Acenaphthylene	0.23	0.92	0.01	3.13	3.4
Acenaphthene	0.24	0.97	0.002	3.14	3.63
Fluorene	0.36	0.96	0.004	3.32	3.71
Phenanthrene	0.42	0.92	0.01	3.39	3.96
Anthracene	0.42	0.88	0.02	3.39	3.98
Fluoranthene	0.44	0.95	0.001	3.4	4.71
Pyrene	0.43	0.98	0.001	3.4	4.86

a. This work; b. [12].

**Table 3:** Statistical significance measured through p values at the US treatment 20 min time point compared with other time points (mixing only, US 10, US 30, after US 10, after US 20, and after US 30). The p values were determined through a multiple comparisons ANOVA (Tukey HSD), a= 0.05.

PAH congener	Mixing only	US 10 min	US 30 min	After US 10 min	After US 20 min	After US 30 min
Phenanthrene	p < 0.001	0.015	p > 0.05	0.006	0.001	0.002
Anthracene	p < 0.001	p > 0.05	p > 0.05	0.001	p < 0.001	p < 0.001
Fluoranthene	0.01	p > 0.05	p > 0.05	p > 0.05	0.023	0.05

Due to their hydrophobic nature, PAHs are preferentially sorbed to the fiber coating rather than remaining in solution. Consistent with previous studies, our results show good linear agreement between the determined $K_{SPME10}$ and $K_{\mathrm{OW}}$ values [12], [34]. For higher molecular weight PAHs, the values we observed are lower than the reported comparison between $K_{\mathrm{SPME}}$ and$K_{\mathrm{OW}}$, likely due to not attaining equilibrium.

### 3.3 [PAH] before, during, and after US treatment

To determine how US affected aqueous PAHs concentrations from PAH contaminated sediment exposed to ultrasound, SPME fibers were used to assess the PAH concentrations in the aqueous phase before, during ultrasonic exposure, and immediately after ultrasonic exposure. A fiber was first exposed to the sediment for 10 min under mixing only conditions. A second fiber was then inserted right after the first one was removed, and US immediately commenced. After the initial 10 min of US, the fiber was replaced. This process was repeated two more times for a total of 30 min of US treatment. When the fiber exposed during the third 10 min of US was removed, a fresh fiber was replaced, and US was immediately ceased. The same process of removal and replacement every 10 min was repeated for a total of 30 min after US, under mixing only conditions. In total, seven exposures were sequentially compiled to develop a change over time, totaling 70 min: mixing only (10 min), US treatment (30 min), mixing only after US treatment (30 min). In addition, in one set of experiments, the same process of exposing fibers for 10 min to sediment slurries was conducted, with mixing but in the absence of ultrasound. Four fiber exposures for 10 min each were also conducted for a total of 40 min under mixing only.

**Fig. 4:**
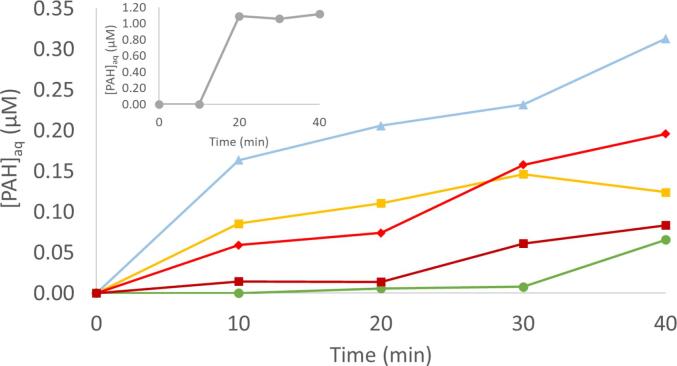
Aqueous PAH concentrations during 40 min mixing only experiment using SPME fibers. Little Scioto sediment slurry concentration = 30 g L^−1^. Fiber removed and fresh conditioned fiber inserted at start of each 10 min period (t = 10, 20, 30, 40). Mixed on shake table at 125 RPM. Reacting solution temperature = 32 ± 3 °C.

**Fig. 5:**
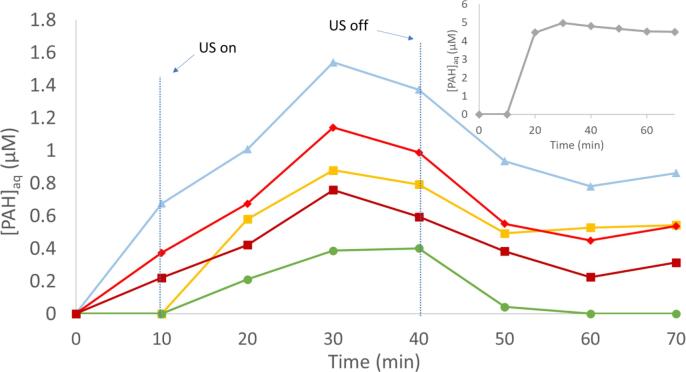
Aqueous PAH concentrations during mixing only (t = 10), during US (t = 20, 30, 40), and after US (t = 50, 60, 70) using SPME fibers. Sediment concentration = 30 g L^−1^. Fiber was removed after every 10 min time interval and a fresh fiber inserted. Mixed before, during and after US on shake table at 125 RPM. Reacting solution temperature = 32 ± 3 °C.

This evolution of aqueous phase PAH concentrations with mixing only and with the addition of ultrasound is shown in Fig. 4, Fig. 5, respectively. As shown in Fig. 4, for mixing only, apart from acenaphthene, the PAH congeners show a slow increase in aqueous PAH concentrations as mixing proceeds. For Fig. 5, a similar increase in aqueous PAH concentrations is observed at 10 min with mixing only. However, during US a larger increase in aqueous concentrations is observed, including a peak in aqueous PAH concentrations. Acenaphthene, the lightest molecular weight PAH quantified, does appear to reach a similar concentration the during mixing only and with US conditions. However, with mixing only it occurs after a 20 min contact time of the fiber. During US, this concentration appears to be reached in 10 min with US on, indicating that US enhances mass transfer of PAHs from sediment into solution, consistent with other studies [37], [42]. For all quantified heavier molecular weight PAHs, the mass released into aqueous solution was higher during US than mixing alone, demonstrating that the release is not due to mixing.

Comparing [PAH] at the 20 min time point of ultrasonic treatment, the measured concentrations of phenanthrene, anthracene, and fluoranthene were higher than in the mixing only condition (Table 3). This enhanced desorption during sonication is consistent with many other studies showing this effect [8], [14], [20], [52]. Treatment may cause PAHs embedded in the sediment to be subjected to acoustic microstreaming which can reach pore spaces and facilitate diffusional mass transfer [46]. This fragmentation effect may even be extended to any sorbed PAHs present, causing them to be more effectively mixed into the system and improving desorption into the water, resulting in higher aqueous concentrations during sonication.

When comparing PAH concentrations during US with after US, Fig. 5 shows higher concentrations during US and lower aqueous phase PAH concentrations after US for all PAHs. Fig. 5 is an average of three experiments, but due to heterogeneity between sediment samples, error bars were not included. The observed trend of higher PAH concentrations during US and lower PAH concentrations after US is consistent among experiments. See SI for graphs from individual experiments.

Degradation of PAHs by ultrasound has been observed in sediment slurries [37], [43]. However, when US is turned off, the total amount of PAHs will no longer be degraded. Because of this, we can infer that there is no change in total PAHs in the system (aqueous and sediment phases). Thus, 40 min and 50 min time points in Fig. 5 have the same total PAHs in the system. Therefore, after sonication, the decrease in aqueous concentration suggests that PAHs resorb to the sediment to some degree.

The resorption is more pronounced for the more hydrophobic compounds with a decrease in concentration of nearly half for phenanthrene when US is off. Table 3 shows the decrease is statistically significant for phenanthrene, anthracene and fluoranthene. Understanding the concentrations during treatment and observing resorption of PAHs onto sediment is an important step in the design and implementation of a successful *in-situ* method of US facilitated release and degradation of PAHs. Particularly, it suggests that if US is used primarily to enhance mass transfer, the treatment of the released PAHs should occur during US as opposed to after US is stopped. This result also suggests that the system during ultrasound has an altered sorption equilibrium condition compared to when ultrasound is terminated.

Table 4 shows the aqueous concentrations (C_w_) measured after 10 min of mixing, after 30 min of US and 30 min after US was terminated. We also report the aqueous solubility of PAH congeners and a calculated equilibrium aqueous concentration from partitioning equations:.

(2) $$K_{d}=f_{\mathrm{OC}}K_{\mathrm{OC}}$$

and

(3) $$K_{d}=\frac{C_{S}}{C_{e}}$$

where $f_{\mathrm{OC}}$ is the fraction of organic carbon on the sediment, K_OC_ is the organic carbon partitioning coefficient [51] and C_S_ is the congener concentration on the sediment before treatment for mixing only and after 30 min of US treatment for US experiments. Using reported K_OC_ values [51] and the $f_{\mathrm{OC}}$ value measured for this sediment reported in Table 1, we calculated K_d_ values for each PAH. K_d_ was then used to calculate C_e_. C_e_ from Eq. (3) yields an equilibrium aqueous PAH concentration that is dependent on the sediment concentration. K_d_ is a ratio of sediment to aqueous concentration, therefore is dependent on the amount of PAHs on sediment. With high C_S_, C_e_ may be calculated to be larger than the reported water solubility. Thus, at equilibrium, the aqueous concentration expected in solution for each congener will be the smaller value of the water solubility and the equilibrium concentration expected, calculated from partitioning, C_e,exp_. We report this as C_Wmax,exp_ in Table 4.

**Table 4:** Aqueous PAH concentrations (C_w_) measured from SPME fibers during 10 min mixing, 30 min US, and 30 min after US was stopped and measured from liquid-liquid extraction following treatment. Expected aqueous concentration (C_e,exp_) calculated using K_OC_, f_OC_, and K_d_ values. Expected maximum PAH aqueous concentrations (C_W,max exp_) are smaller of water solubility and C_e,exp_.

PAH congener	C_W_, mg/L				Water solubility mg/L^a^	C_e,exp_ mg/L	C_W,max exp_ mg/L
	Mixing only 10 min	US on 30 min	US off 30 min	Recovered from water after US			
Naphthalene	N.D.	N.D.	N.D.	N.D.	31.7	N.D.	N.D.
Acenapthylene	N.D.	N.D.	N.D.	0.011	3.93	0.020	0.020
Acenaphthene	N.D.	0.74	0.70	N.D.	16.1	0.0029	0.0029
Fluorene	N.D.	0.13	0.090	0.0029	1.98	0.0020	0.0020
Phenanthrene	0.12	0.25	0.15	0.017	1.29	0.072	0.072
Anthracene	N.D.	0.071	N.D.	0.057	0.073	0.086	0.073
Fluoranthene	0.075	0.20	0.11	0.10	0.26	0.093	0.093
Pyrene	0.045	0.12	0.064	0.075	0.135	0.046	0.046

N.D. = not detected; a. From [51].

As shown in Table 4, during ultrasound, with the exception of anthracene, all compounds have aqueous concentrations higher than the expected equilibrium concentration. The least soluble PAHs quantified (anthracene, fluoranthene, pyrene) appear to reach the aqueous solubility during ultrasound and surpass the aqueous concentration expected from equilibrium sorption using K_d_. For the more soluble PAHs (acenaphthene, fluorene, phenanthrene), observed aqueous concentrations are much higher than predicted from equilibrium sorption using K_d_. Thus, US facilitates the dissolution of these PAHs into water, above that predicted by equilibrium.

Aqueous concentrations of PAHs decrease over the first 10 min after ultrasound is ceased and then stabilize. The concentrations of more soluble PAHs, while lower than measured while US was on, still remain higher than the C_e,exp_ concentrations. The concentrations appear to decrease back to equilibrium concentrations expected from partitioning for anthracene, fluoranthene, and pyrene when US was ceased. The decrease of aqueous concentration is larger after US is ceased for the less soluble and more hydrophobic compounds.

The decrease in the aqueous concentration after ultrasound compared to during ultrasound is stopped suggests a different equilibrium condition during ultrasound compared to after ultrasound. To our knowledge, this effect has not been shown previously. Li et al. [31], using a 24-hour equilibrium time, observed different sediment-crude oil adsorption isotherms with ultrasound compared to mixing. He et al. [22] investigated the release of mercury from aluminum oxide (a simple model sediment). This work showed an increase in mercury released into solution followed by a decrease at longer sonication time. Moreover, the Al or Si concentrations reached levels well-above the solubility limit of the mineral oxides present [22], [32]. These previous studies did not investigate in detail the stability or reasons behind the non-equilibrium concentrations reached for Al or Si. XRD measurements of sonicated particles did not show any bulk mineral changes [22], [52] but decreases in particle size has been observed by many (examples: [15], [32], [43]. In addition, organic matter released from the sediment has been linked to contaminant release [23] and been shown to resorb to sediment particles with longer sonication time [24], [43]. While these experiments were all conducted by analyzing contaminants and bulk properties after ultrasound is stopped, these experiments show that resorption occurs. The study of Pee et al. [43] was conducted on PAH contaminated sediment from the same location as this work indicating that those results are relevant to this work. While these studies suggest that TOC release may be linked to PAH release and resorption, none of this previous work demonstrates that the amount desorbed during ultrasound is more than after ultrasound. Our result indicates a non-equilibrium condition during ultrasound. While we tested only one sediment, sorption of hydrophobic compounds such as PAHs to sediment will depend on the fraction of organic matter and the hydrophobicity of the compound. The range of hydrophobicities of the PAHs tested and the similarity in the resorption for the majority of them suggest that this effect may be observed beyond the sediment tested in this study.

Our result is important when designing a remediation process for contaminated sediment using ultrasound. A remediation process for contaminated sediment using ultrasound can be designed in multiple ways. One way would be to apply ultrasound and then remove the supernatant to treat the aqueous concentration released. Previous work has yielded information relevant for this sort of a design. An alternate way to design a remediation process would be to apply ultrasound at the same time as a treatment to treat the aqueous concentration during sonication. Our work reveals that this alternate design would result in higher concentrations in the aqueous phase, by as much as a factor of 2, which may considerably alter the cost and treatment times required compared to treating the aqueous stream after ultrasound.

### 3.4 PAHs extraction from sediment after US

Following treatment, PAHs were extracted from the sediment. The resulting levels of PAHs extracted after treatment compared to before treatment are shown in Table 5. Release of PAHs during US makes them accessible for degradation, which appears to have occurred for a portion of PAHs while in the aqueous phase. PAHs degradation in the presence of US has been documented [37], [43], [44]. Furthermore, a mixture of PAHs results in a greater observed degradation rate in the presence of US than individual PAHs.

**Table 5:** PAHs determined from total solvent extraction from sediment before and after US treatment and total solvent liquid–liquid extraction from water recovered after US treatment.

PAH congener	Amount, µmol			Calculated degradation^c^
	Before US^a^	After US^b^	Recovered from water^a^	
Naphthalene	N.D.	N.D.	N.D.	
Acenaphthylene	0.72 ± 0.08	0.1 ± 0.3	0.004 ± 0.003	
Acenaphthene	0.9 ± 0.2	0.02 ± 0.04	N.D.	
Fluorene	0.8 ± 0.2	0.03 ± 0.04	0.001 ± 0.001	
Phenanthrene	2.4 ± 0.3	0.6 ± 0.3	0.005 ± 0.003	1.8
Anthracene	3.8 ± 0.7	1.2 ± 0.6	0.02 ± 0.01	
Fluoranthene	6.7 ± 0.7	2.3 ± 1.1	0.03 ± 0.02	
Pyrene	7.5 ± 0.8	1.4 ± 0.6	0.02 ± 0.01	4.1

a determined from triplicate analysis.

b determined from quintuplicate analysis.

c degradation rate constant from Pee [42].

As US affects both sediment and water, a higher release into the aqueous phase makes PAHs accessible for degradation. The concentration which is released into the aqueous phase is a very small portion of the total (sediment and aqueous) concentration present. However, US promotes the release of sorbed PAHs which surpass equilibrium concentrations, replenishing the aqueous concentration while simultaneously degrading the PAHs. Comparison of the total mass (µmol) quantified with the total mass (µmol) released at the peak of aqueous concentration (Table 5) shows that for lower molecular weight PAHs, like acenaphthene, which have a higher solubility limit, more is released into the aqueous phase, resulting in greater accessibility for degradation. However, for the heavier molecular weight PAHs, the decrease in measured PAH levels after US is not as large. The degradation using first-order degradation rate constants for phenanthrene and pyrene determined by Pee [42] using similar conditions (k = 0.01, 0.02 min^−1^, respectively), reasonably explain the decrease in sediment concentration.

## 4 Conclusion

Application of US to PAH contaminated sediment has an apparent effect on resulting aqueous phase PAH concentrations during ultrasound, as measured through SPME. Measured aqueous phase phenanthrene, anthracene, and fluoranthene concentrations were statistically higher than concentrations after US was ceased. For all compounds, there is an obvious reduction in concentration after ultrasound is stopped. Therefore, studies testing US for sediment remediation may under report the effect of ultrasound due to the nature of how the contaminants are measured after treatment. These results have ramifications in determining the effectiveness of ultrasound for remediation. Typical analytical techniques extract the target contaminant after ultrasound is ceased. After as little as 10 min, the aqueous concentration is lower than that achieved during ultrasound. While it is well-known that ultrasound accelerates mass transfer when compared to mixing, to our knowledge, this is the first report of higher than equilibrium release during ultrasound that decreases within 10 min after ultrasound is stopped. This work suggests using SPME will assist in understanding the effectiveness of ultrasound for desorption of target contaminants from solids and can be adapted to a variety of sediments and contaminants and used to more accurately assess the effectiveness of ultrasound as an in-situ remediation technology. Moreover, this method reveals that ultrasound will be most effective when combined with degradation or a method to sorb released contaminants during ultrasound rather than after an ultrasonic treatment.

## CRediT authorship contribution statement

**Danielle S. Kohan:** Conceptualization, Methodology, Data curation, Formal analysis, Visualization, Writing – original draft. **Roman P. Lanno:** Conceptualization, Methodology, Formal analysis. **Linda K. Weavers:** Supervision, Conceptualization, Resources, Writing – review & editing.

## Declaration of Competing Interest

The authors declare that they have no known competing financial interests or personal relationships that could have appeared to influence the work reported in this paper.
