# Supplementary material for: Using solid-phase microextraction during ultrasound reveals higher aqueous PAHs release from contaminated sediment
Source: Ultrason Sonochem. 2022 Mar 12;85:105981. doi: 10.1016/j.ultsonch.2022.105981 (PMC8938626; doi:10.1016/j.ultsonch.2022.105981)
Supplement: Supplementary data 1 [file mmc1.docx]

**Supplemental Information**

Using Solid-Phase Microextraction during Ultrasound Reveals Higher Aqueous PAHs Release from Contaminated Sediment

Danielle S. Kohan ^‡^, Roman P. Lanno^‡§^, Linda K. Weavers^‡†^*

^‡^Environmental Science Graduate Program, The Ohio State University, Columbus, Ohio 43210, United States

^§^Department of Evolutionary Ecology and Organismal Biology, The Ohio State University, Columbus, OH 43210, United States

^†^Department of Civil, Environmental, and Geodetic Engineering, The Ohio State University, Columbus, Ohio 43210, United States

*To whom correspondence should be addressed. Phone: (614) 292-4061; Fax: (614) 292-3780; email address: [weavers.1@osu.edu](mailto:weavers.1@osu.edu).

**Supplemental Information: Table of Contents**

**Text S1.** Fiber conditioning and control experiments.

**Text S2**: Fiber extraction method.

**Text S3**: Temperature control protocol.

**Figure S1.** Schematic of SPME fiber extraction protocol.

**Figure S2**. Phenanthrene calibration curve (top). Middle and bottom represent portions of chromatograms from samples taken at t=30 min and t=60 min, respectively. Phenanthrene retention time is 7.04 min. Samples analyzed were taken using SPME fibers during ultrasonic treatment at 430 W L^-1^. Sediment concentration = 30 g L^-1^. Fresh conditioned fiber inserted prior to each measurement. Mixing before, during and after US with shake table at 125 RPM. Reacting solution temperature = 32 ± 3ºC.

**Figure S3**. Portions of chromatograms of blanks run during GCMS analysis during the same sequence as chromatograms shown in Fig S2. Top represents first sample run in sequence, middle represents blank run after experiment extraction samples but before calibration standard samples, bottom represents final sample after calibration samples.

**Figure S4.** Phenanthrene carryover samples analyzed during the same experiment and GC/MS sequence as in Figs S2 and S3. Phenanthrene retention time 7.04. LOD and LOQ were estimated as clear and defined peaks with signal to noise ratios of 3 and 10, respectively. Carryover method described in Fig S1. No carryover was detected for these samples, carryover for all experiments was <10%.

**Figure S5.** Individual experiments measuring aqueous PAH concentrations during mixing only (t=10), during US (t=20, 30, 40), and after US (t=50, 60, 70).

**Text S1.** Fiber conditioning and control experiments

Before every experiment, fibers were serially conditioned in three 7.5 mL aliquots of 1:1 hexane/acetone for 10 minutes each on a shake table (New Brunswick Scientific, C1 Platform Shaker) at speed setting 30. Newly constructed fibers also underwent one cycle of exposure to aqueous solution of PAHs and subsequent desorption to prepare the PDMS coating for regular use. To test the reproducibility of our methods, two different fibers were exposed to the same concentrations of aqueous PAH solutions, and one fiber was exposed three different times for a total of five exposures. Error was determined to be less than 10%. The average of these exposures was used as the expected fiber concentration for that starting aqueous concentration.

Additionally, a fiber durability test was conducted to measure how exposure to sediment particles in the slurry affected the fiber coating. A fresh fiber was exposed to 30 g L^-1^ sediment slurry and sonicated for 10 min. The fiber was desorbed after sonication and exposed to a fresh aqueous PAH solution at the same concentration that was used in the reproducibility determination. The fiber was desorbed and the concentration was analyzed. This process of sonication, desorption, exposure, and desorption was repeated on the same fiber a second time. Compared with the established expected concentration of an undamaged fiber, a fiber that underwent three or more 10 min exposures to US appeared to be damaged as was evidenced by lower PAH concentrations extracted from the fiber. Concentrations determined by fibers that had been sonicated once or twice were within 10% or less than mean of unsonicated fibers, with the exception of naphthalene. A different fresh fiber was also exposed to a sediment slurry for 20 continuous min and the resulting damage was tested. The 20 min exposure also resulted in significantly lower PAH concentrations extracted from the fiber. Additionally, in some trials the fiber broke during treatment. Thus, fiber exposures were limited to 10 min increments and a fiber was replaced after every other 10 min exposure to ensure that the resulting concentrations were not affected by damage to the fiber coating or fiber itself.

**Text S2**: Fiber extraction method

After conditioning, the fiber was immersed in aqueous PAH solution or sediment slurry to equilibrate with the aqueous PAH solution under treatment conditions. As shown in Figure S1, after exposure to the aqueous PAH solution or sediment slurry, the fiber was successively desorbed into two 5mL aliquots of 1:1 hexane/acetone on the shake table which were mixed together for a final volume of 10mL. Subsequently, 0.9 mL was transferred to a 1.5 mL GC vial for analysis. As a carryover measure, the fiber was exposed to a final 5mL aliquot. This third 5mL aliquot was quantified through the GC/MS; however, the results were only used to ensure that the first two aliquots were sufficient in capturing all PAHs sorbed to the fiber. Throughout experiments, <10% PAHs were detected in carryover samples.


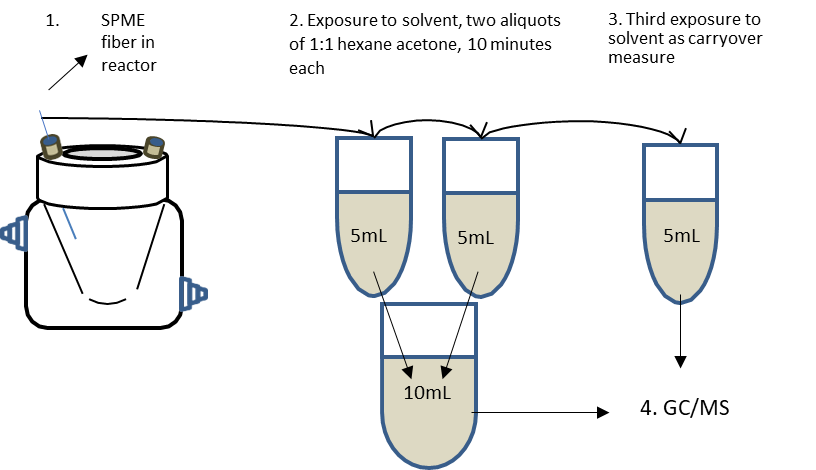


**Figure S1**. SPME fiber extraction protocol. After an SPME fiber was exposed to a PAH contaminated sediment slurry, it was immersed in two serial 5mL aliquots of 1:1 hexane acetone for 10 minutes each on a shake table. These two aliquots were mixed together and analyzed. The fiber was then immersed in a third aliquot to ensure that the first two aliquots were successful in removing the PAHs from the fiber.

**Text S3**: Temperature control protocol

During ultrasonic treatment, the release of heat due to cavitation bubble implosion causes the temperature of the system to increase. Even when using a water bath to circulate water at a consistent temperature through the water jacket surrounding the reactor, a ~10 ℃ increase in water temperature was observed when investigated with a thermocouple. To control for this, the water bath was set to 32 ℃ for the mixing only condition, prior to US treatment. Before US treatment commenced, the water bath was turned down to 20 ℃. Finally, 2.5 minutes before the completion of the US treatment, refrigeration control on the water bath was turned off and the temperature was set back to 32 ℃. At the moment directly before US was turned off, the temperature in the reactor was 33 ℃ which, when US was stopped, dropped to 27 ℃ and then returned to 32 ℃ within 4 minutes of US being off. Overall, this protocol maintained the temperature at 32ºC ± 3ºC in the reactor.


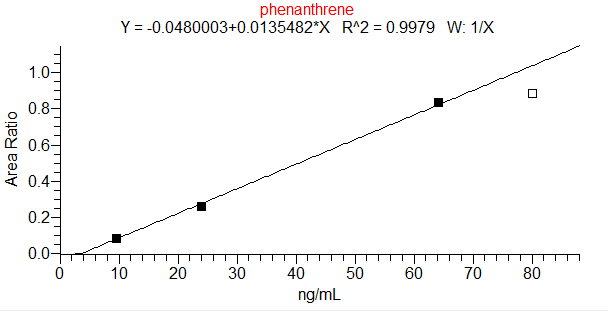

**Figure S2**. Phenanthrene calibration curve (top). Middle and bottom represent portions of chromatograms from samples taken at t=30 min and t=60 min, respectively. Phenanthrene retention time is 7.04 min. Samples analyzed were taken using SPME fibers during ultrasonic treatment at 430 W L^-1^. Sediment concentration = 30 g L^-1^. Fresh conditioned fiber inserted prior to each measurement. Mixing before, during and after US with shake table at 125 RPM. Reacting solution temperature = 32 ± 3ºC.

**Figure S3**. Portions of chromatograms of blanks run during GCMS analysis during the same sequence as chromatograms shown in Fig S2. Top represents first sample run in sequence, middle represents blank run after experiment extraction samples but before calibration standard samples, bottom represents final sample after calibration samples.


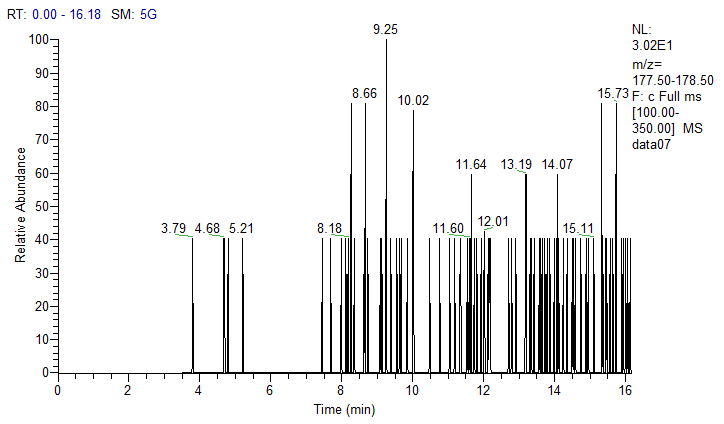


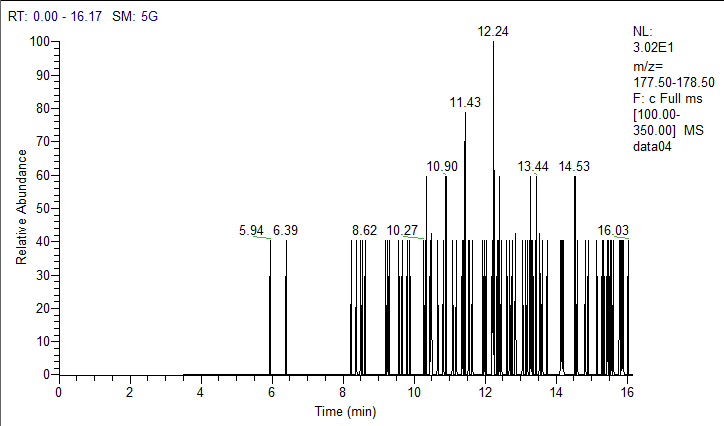


**Figure S4.** Phenanthrene carryover samples analyzed during the same experiment and GC/MS sequence as in Figs S2 and S3. Phenanthrene retention time 7.04. LOD and LOQ were estimated as clear and defined peaks with signal to noise ratios of 3 and 10, respectively. Carryover method described in Fig S1. No carryover was detected for these samples, carryover for all experiments was <10%.


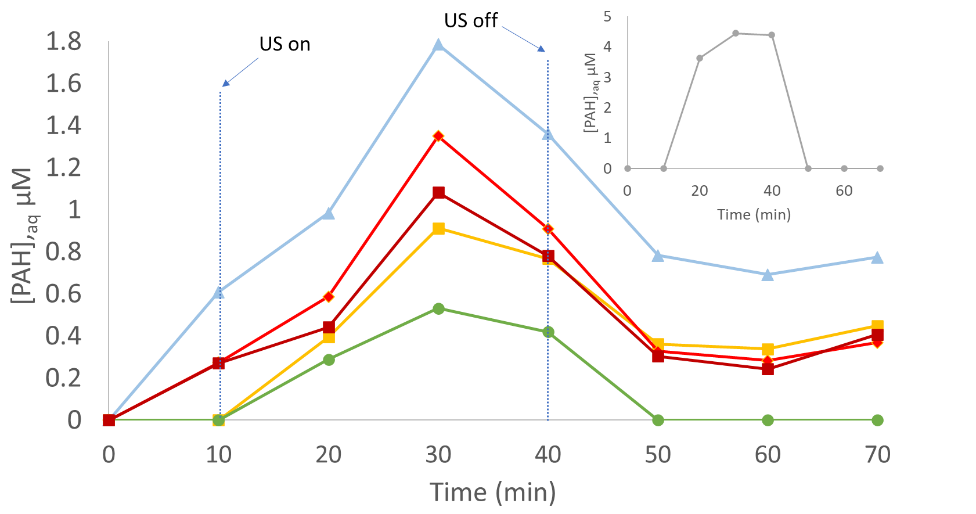

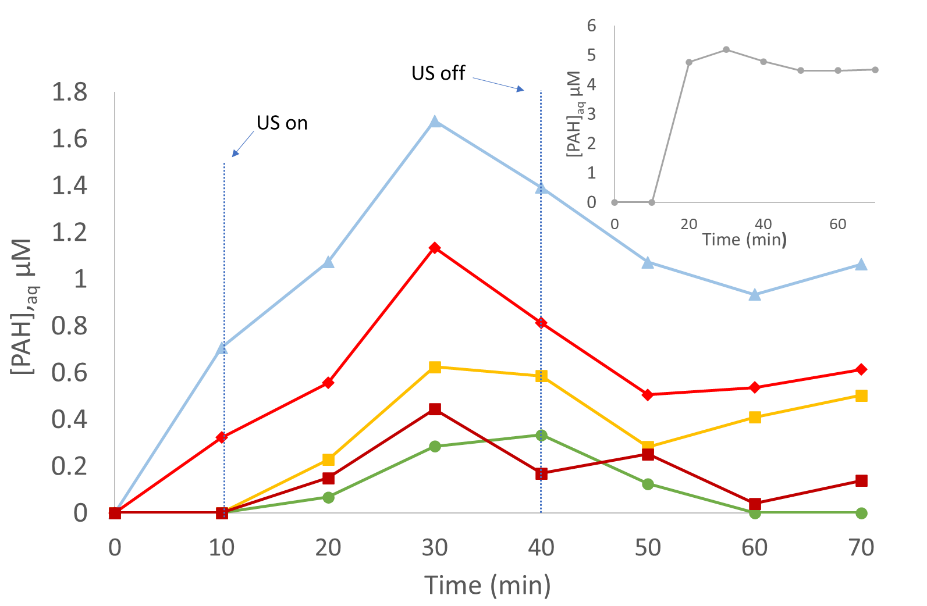

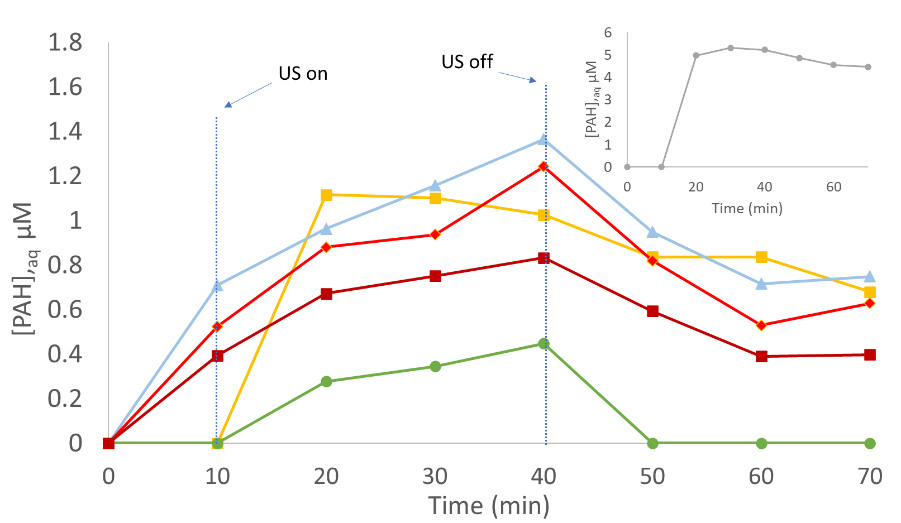
**Figure S5.** Individual experiments measuring aqueous PAH concentrations during mixing only (t=10), during US (t=20, 30, 40), and after US (t=50, 60, 70). Sediment concentration = 30 g L^-1^. Fresh conditioned fiber inserted at start of each 10 min. period. Mixing before, during and after US with shake table at 125 RPM. Reacting solution temperature = 32 ± 3ºC.
